# Field Test and Validation of the Multiplier Measles, Mumps, Rubella, and Varicella-Zoster Multiplexed Assay System in the Democratic Republic of the Congo by Using Dried Blood Spots

**DOI:** 10.1128/mSphere.00112-19

**Published:** 2019-08-14

**Authors:** Stephen G. Higgins, Nicole A. Hoff, Adva Gadoth, Andrew Fusellier, Patrick Mukadi, Vivian Alfonso, Christina Randall, Hayley Ashbaugh, Melanie Poncheri, Reena H. Doshi, Sue Gerber, Roger Budd, Robert Wolfert, Russell Williams, Emile Okitolonda-Wemakoy, Jean-Jacque Muyembe-Tamfum, Anne W. Rimoin

**Affiliations:** a Dynex Technologies Inc., Chantilly, Virginia, USA; b Department of Epidemiology, University of California, Los Angeles, Fielding School of Public Health, Los Angeles, California, USA; c National Institute of Biomedical Research (INRB), Kinshasa, Democratic Republic of the Congo; d Faculty of Medicine, University of Kinshasa, Kinshasa, Democratic Republic of the Congo; e Bill and Melinda Gates Foundation, Seattle, Washington, USA; f UCLA-DRC Research Program, Kinshasa, Democratic Republic of the Congo; g Kinshasa School of Public Health, University of Kinshasa, Kinshasa, Democratic Republic of the Congo; University of Maryland School of Medicine

**Keywords:** Democratic Republic of the Congo, MMRV, assay validation, multiplex assay

## Abstract

Here we describe baseline validation studies and field performance of a research-use-only chemiluminescent multiplex serology panel for measles, mumps, rubella, and varicella-zoster virus used with dried blood spots in support of the 2013–2014 Democratic Republic of the Congo Demographic and Health Survey. Characterization of the panel using U.S. FDA-cleared commercial kits shows good concordance for measles, mumps, rubella, and varicella-zoster with average sensitivity across assays of 94.9% and an average specificity of 91.4%. As expected, performance versus available standards validated for plaque-reduction assays does not provide a 1:1 correspondence with international units and yet demonstrates excellent linearity (average Hill’s slope = 1.02) and ∼4 logs of dynamic range. In addition, for the four assays, the multiplexed format allowed for inclusion of three positive and two negative controls for each sample. A prototype Dynex Multiplier chemiluminescent automated immunoassay instrument with a charge-coupled device camera provided a rugged and robust processing and data acquisition platform. Performance of a multiplex instrument for serological testing in a substantially resource-limited environment shows excellent reproducibility, minimal cross-reactivity, and a clear discrimination between specific assays and should be considered a viable option for future serosurveys.

**IMPORTANCE** The critical evaluation of immunization programs is key to identifying areas of suboptimal vaccination coverage, monitoring activities, and aiding development of public health policy. For evaluation of vaccine effectiveness, direct antibody binding assay methods, including enzyme immunoassay, enzyme-linked fluorescence assays, and indirect immunofluorescence assay, are most commonly used for detection of IgG antibodies. However, despite their well-demonstrated, reliable performance, they can be labor-intensive and time-consuming and require separate assays for each individual marker. This necessitates increased sample volumes, processing time, and personnel, which may limit assessment to a few key targets in resource-limited settings, that is, low- and middle-income locations where funding for public health or general infrastructure that directly impacts public health is restricted, limiting access to equipment, infrastructure, and trained personnel. One solution is a multiplexed immunoassay, which allows for the detection of multiple analytes in a single reaction for increased efficiency and rapid surveillance of infectious diseases in limited-resource settings. Thus, the scope of the project precluded a full validation, and here we present abbreviated validation studies demonstrating adequate sensitivity, specificity, and reproducibility.

## Abstract (precis)

The critical evaluation of immunization programs is key to identifying areas of suboptimal vaccination coverage, monitoring activities, and aiding development of public health policy. For evaluation of vaccine effectiveness, direct antibody binding assay methods, including enzyme immunoassay, enzyme-linked fluorescence assays, and indirect immunofluorescence assay, are most commonly used for detection of IgG antibodies. However, despite their well-demonstrated, reliable performance, they can be labor-intensive and time-consuming and require separate assays for each individual marker. This necessitates increased sample volumes, processing time, and personnel, which may limit assessment to a few key targets in resource-limited settings, that is, low- and middle-income locations where funding for public health or general infrastructure that directly impacts public health is restricted, limiting access to equipment, infrastructure, and trained personnel. One solution is a multiplexed immunoassay, which allows for the detection of multiple analytes in a single reaction for increased efficiency and rapid surveillance of infectious diseases in limited-resource settings. Thus, the scope of the project precluded a full validation, and here we present abbreviated validation studies demonstrating adequate sensitivity, specificity, and reproducibility.

## INTRODUCTION

The critical evaluation of immunization programs is key to identifying areas of suboptimal vaccination coverage, monitoring activities, and aiding development of public health policy. While the incidence of vaccine-preventable diseases (VPDs) such as measles, mumps, rubella, and varicella-zoster virus (VZV) infection (MMRV) has been substantially reduced in developed countries due to effective immunization programs, much work remains in low- and middle-income countries (LMICs) (1, 2). In LMICs, unreliable infrastructure and inadequate public health systems can lead to gaps in immunization against VPDs, often leaving the poorest and most difficult-to-reach members of the population susceptible to infection (3–5). While most countries have a national immunization program which provides a number of standard routine vaccinations for children and pregnant women, not all countries have introduced vaccines against MMRV into the national immunization framework, and administrative data may not be reliable for determining accurate rates of seroprotection (6). Therefore, serosurveys present an important opportunity for direct and accurate assessment of population-level antigen exposure or susceptibility (7–13) and can provide critical insight into ongoing immunity gaps and operational program efficiency.

For surveillance of infectious diseases and evaluation of vaccine effectiveness, enzyme-linked immunosorbent assay (ELISA) methods, including enzyme immunoassay (EIA) and enzyme-linked fluorescence assay (ELFA), are most commonly used for detection of immunoglobulin G (IgG) antibodies (14). ELISA is considered the gold standard for measuring soluble biomarkers and is widely accepted in clinical practice (15). However, despite their well-demonstrated, reliable performance, ELISAs can be labor-intensive and time-consuming and require separate assays for each individual marker. This necessitates increased sample volumes, processing time, and personnel, which may limit assessment to a few key targets in resource-limited settings. One solution is a multiplexed immunoassay, which allows for the detection of multiple analytes in a single reaction for increased efficiency and rapid surveillance of infectious diseases in limited-resource settings.

While demonstrating good performance, many validated seroprevalence results have been based on serum, plasma, or whole-blood samples (1, 14, 16–18), and further work is ongoing to demonstrate that dried blood spot (DBS) extracts lead to comparable results (19–22). Whole-blood vial collection is much more difficult than DBS due to additional logistical needs and expenses related to sample processing, storage, and cold chain and increased difficulty of obtaining venipuncture blood from children and the elderly (20, 22). Therefore, during large-scale surveillance or screening activities, the collection of DBSs in lieu of wet specimens is common practice in LMICs given that DBS collection is minimally invasive, reduces cost for sample collection, requires less cold-chain management, and may be better accepted by the donor and/or parent (23).

In 2013, Dynex Technologies Inc. (Dynex), Chantilly, VA, developed a research-use-only multiplex chemiluminescent immunoassay panel. While the field test described here utilizes DBSs, the intended use and validation of the assay panel were with serum. The DBS samples, collected during a large-scale, nationally representative serosurvey embedded in the 2013–2014 Democratic Republic of the Congo Demographics and Health Survey (DRC-DHS), were processed on the prototype Dynex Multiplier chemiluminescent automated immunoassay instrument at the Institut National de Recherche Biomédicale (INRB) in Kinshasa, Democratic Republic of the Congo, to test for reactivity against MMRV with the addition of a tetanus test (MMRVT) on the same platform. (The tetanus assay was treated separately from MMRV since it was not included in product development going forward and had additional considerations for validation that were not considered for the MMRV portion of the assay and will be included in a subsequent evaluation. However, the product used for all testing was the MMRVT [measles, mumps, rubella, varicella, and tetanus].) In the context of this study, “field test” refers to samples being collected, stored, and shipped from remote field sites to the INRB, which in light of intermittent 50-Hz electricity supplemented by generators and uninterruptible power supply (UPS) battery backup, onsite water distillation, and limited access to reagents and laboratory consumables is by Western standards resource limited. In order to determine the appropriateness and feasibility of the multiplex assay, we assessed its performance against standard ELISAs for the detection of MMRV IgG antibodies in accordance with the World Health Organization’s Special Program for Research and Training in Tropical Diseases guidance on diagnostic test validation studies (24). The scope of the project precluded a full validation, and here we present abbreviated validation studies demonstrating adequate sensitivity, specificity, and reproducibility. Our goals are threefold: (i) to evaluate the performance of the Dynex MMRV chemiluminescent automated immunoassay compared to standard ELISA kits, (ii) to assess the use of reconstituted DBSs in this assay, and (iii) to demonstrate the utility of multiplex testing to rapidly determine population immunity using field-collected specimens in a resource-limited setting.

## RESULTS

### MMRV cutoff.

**FIG 1:**
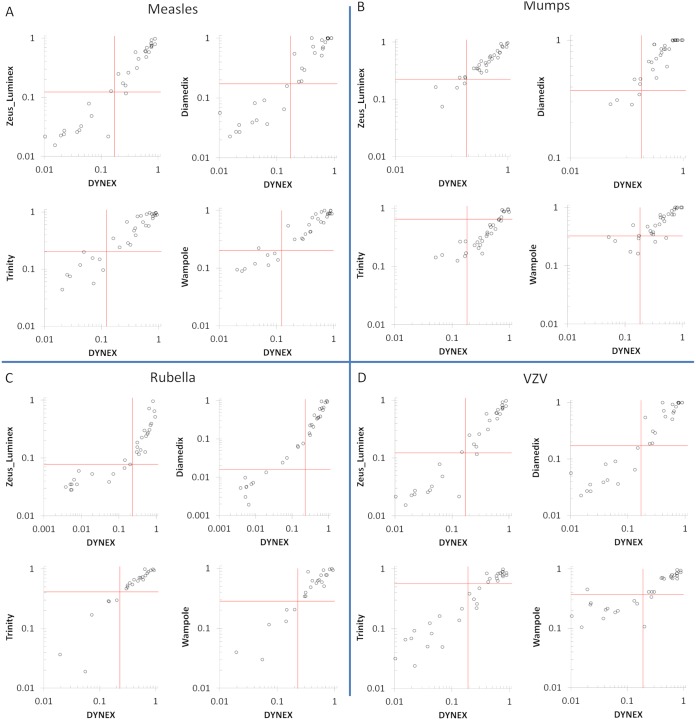
Regression analysis of each FDA-cleared ELISA predicate versus the Dynex system. Each set of results was normalized to facilitate comparisons across platforms. Horizontal red lines represent manufacturers’ cutoffs, and vertical red lines represent the Dynex threshold providing the best fit across all platforms.

The Dynex multiplexed assay cutoff was selected to maximize agreement between all comparators (Table 1). Sensitivity and specificity were calculated assuming that all positive and indeterminate calls for a given comparator represent “true positives” with respect to that comparator and that negative calls represent “true negatives” (Table 2). The average sensitivity is 94.9%, and average specificity is 91.4%. The cutoff for the Trinity mumps kit is quite high versus the other comparators, and thus, specificity in that case is commensurately lower at 58.1%. By excluding that point, the average specificity increases to 93.6%. The average coefficient of determination (*R*^2^) value is 0.86. Regression plots are shown in Fig. 1A to D. Signals have been scaled to the highest value in each data set, with the prototype Dynex Multiplier with MMRV multiplexed 96-well format assay on the *x* axis in each plot. The red lines represent the cutoff for each kit, and the relatively high mumps cutoff for the Trinity kit can be clearly visualized (Fig. 1B).

**TABLE 1:** Limits of detection and linearity^*a*^

Virus	NIBSC standard	Detection limit, mIU	Linearity Hill slope	Assay response	
				Seroprotection	LOQ
Measles	97/648	0.54	0.96	0.141	0.03
Mumps	NA, response to 97/648	0.44	1.02	0.5	0.28
Rubella	RUBI-1-94	3.84	1.10	0.373	0.27
VZV	W1044	0.21	1.02	0.341	0.1

a Serial dilutions of international standards for measles, rubella, and VZV were analyzed, showing good linearity and dynamic range for all assays. In the absence of an international standard for mumps, response to the measles standard is shown. NA, not available. It should be noted that measles standard has not been validated for immunoassays, and these values are relative. Seroprotection cutoffs are represented as assay score, the ratios of sample assay score to in-plate PPC, a blend of 5 North American donors that serves as a reference point on each plate. Limit of quantitation (LOQ) here represents the detection of specific assay response as assay score versus PPC.

**TABLE 2:** Sensitivity and specificity versus commercial ELISA kits^*a*^

Virus and kit name	Value for Multiplier vs commercial kit		
	% sensitivity	% specificity	*R*^2^
Measles			
Diamedix	100.0	81.8	0.76
Wampole	95.8	100.0	0.88
Trinity	100.0	100.0	0.85
Zeus^*b*^	100.0	100.0	0.83
Mumps			
Diamedix	89.3	100.0	0.74
Wampole	95.8	75.0	0.83
Trinity	100.0	58.1	0.91
Zeus	89.3	100.0	0.93
Rubella			
Diamedix	78.3	100.0	0.90
Wampole	100.0	100.0	0.89
Trinity	90.0	93.8	0.95
Zeus	90.9	100.0	0.72
VZV			
Diamedix	100.0	100.0	0.89
Wampole	89.5	84.6	0.88
Trinity	100.0	77.3	0.90
Zeus	100.0	91.7	0.94

a The prototype Dynex Multiplier with MMRV multiplexed 96-well format assay was compared to three conventional ELISAs and one multiplexed Luminex kit using a 32-member reference panel selected to give a range of responses. Sensitivity and specificity were calculated by considering all equivocal results as positive.

b Zeus, multiplexed Luminex kit.

### DBS control extractions in the Democratic Republic of the Congo.

**FIG 2:**
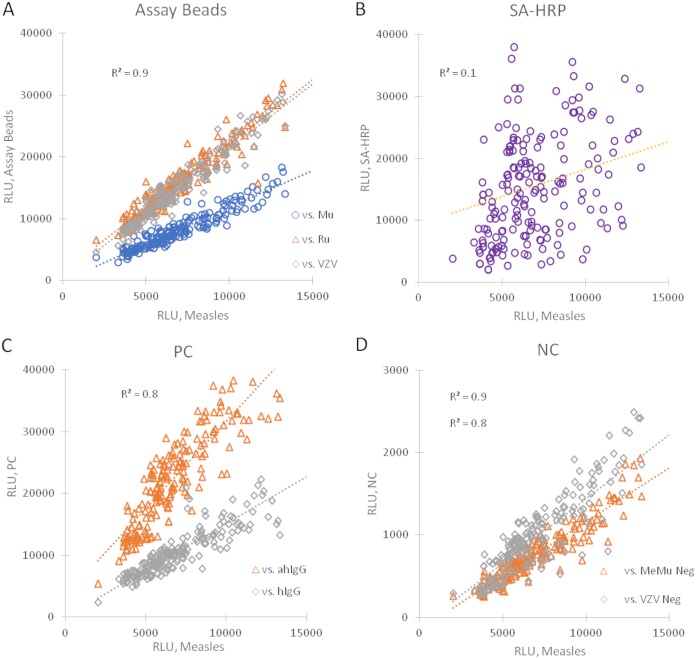
Regression analysis of in-well measles versus assay and control beads in response to pooled positive-control (PPC) extracts. These data were collected during the dried blood spot (DBS) serosurvey. All correlations are relative light units (RLU) of assay bead versus measles RLU on the *x* axis. Assay-specific run-to-run RLU for all assays showed substantial variation, with %CV in excess of 35%. Regression analysis of measles versus specific assay beads shows that, relative to one another, all assay partners within a well respond similarly (A) and so demonstrate an effective control of run-to run variation. Except for the SA-HRP bead (B), which is independent of both extraction and processing, these relationships are seen versus the in-well positive (C) and in-well negative (D) controls, indicating good control of run-to-run variation via intrawell and intraplate positive controls.

**FIG 3:**
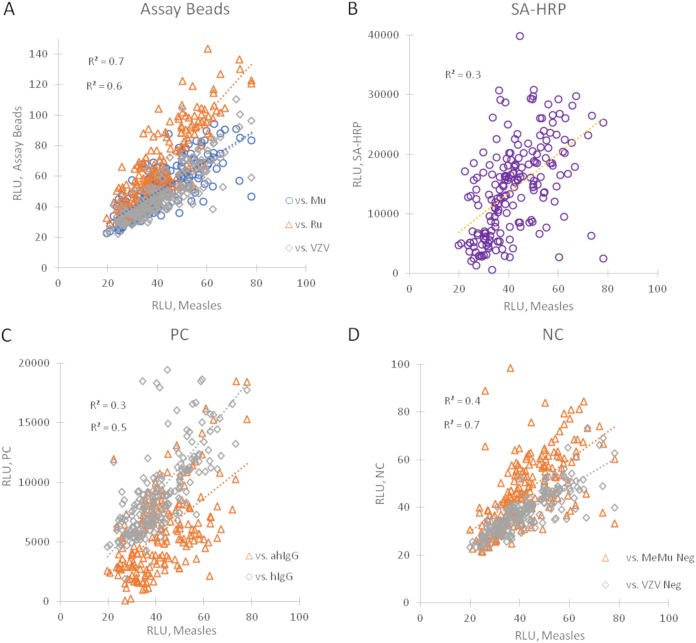
Regression analysis of in-well measles versus assay and control beads in response to IgG-stripped negative-control (NC) extracts. NC extraction regression plots of measles RLU versus assay and control RLU. Compared to PPC regressions seen in Fig. 2, IgG-stripped NC extractions demonstrate lower intensity but similar relationships between specific assay beads (A), SA-HRP positive-control beads (B), in-well positive-control beads (C), and in-well negative controls (D). These relationships further demonstrate that substantial run-to-run variation can be controlled for by PPC and NC extractions.

**FIG 4:**
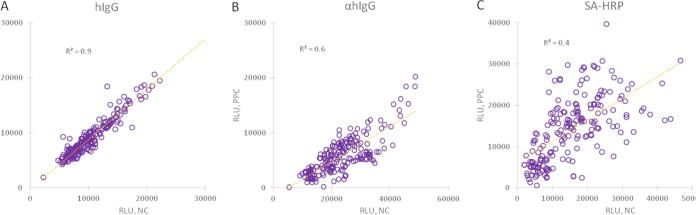
Intraplate correlations of in-well control assays. Comparison of independent extractions of pooled positive control (PPC) and IgG-stripped negative control (NC) within each plate indicates substantial agreements for the hIgG assay (A). This assay responds directly to the detection conjugate and is independent of extraction, giving confidence that run-to-run variability is observed independently across each assay plate. The anti-hIgG assay (B), being dependent upon the extraction process itself, shows a reduced *R*^2^ but for a given assay plate indicates that extractions are treated equivalently. The SA-HRP bead (C) is independent of all processing steps and shows minimal correlation.

As described in the Materials and Methods section, each plate utilized two DBS extraction controls, a pooled positive control (PPC) which provides a reference point for specific assay response and an IgG-stripped serum negative control (NC). In the absence of extensive validation, these controls can serve as independent reference points for critically assessing reproducibility of (i) DBS extraction, (ii) intrawell variability, (iii) intraplate variability, and (iv) interplate variability. Percent coefficients of variation (%CV) of greater than 35% are seen for all assays in PPC extractions, but in-well *R*^2^ correlations for the PPC assays range from 0.9 to 1.0 (Table 3) with *n* = 196, indicating that, relative to each other, intrawell assay relative light units (RLU) was constant. *R*^2^ of intrawell assay RLU relative to the control beads averaged 0.9 and 0.8 for measles and mumps (MeMu)- and VZV-negative controls and 0.7 and 0.8 for anti-human IgG (hIgG)- and hIgG-positive controls, respectively (Fig. 2). The *R*^2^ versus the streptavidin-horseradish peroxidase (SA-HRP) bead, which is independent of the extraction and assay processes, averages 0.1. These relationships are likewise observed for NC extractions, reflecting substantial consistency between independent extractions within a plate (Fig. 3). This is further supported by a substantial correlation of the anti-hIgG assay, measuring extracted human IgG, with *R*^2^ of 0.9 between PPC and NC extractions (Fig. 4). The hIgG control bead, reflecting run-to-run variations of the detection antibody, has a substantially lower *R*^2^ of 0.6.

**TABLE 3:**
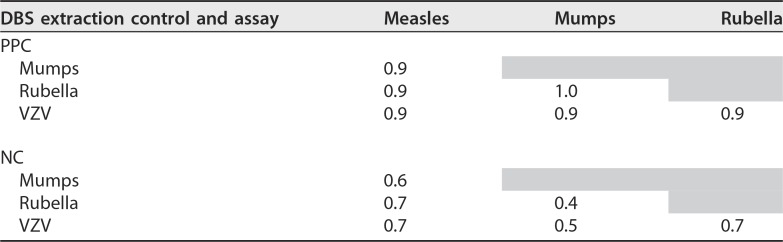
Correlations between DBS control extractions^*a*^

a A wide range of relative signal intensities of specific assay beads for both PPC and IgG-stripped negative control (NC) were observed in the 192 independent runs that compose this data set. However, an *R*^2^ of ≥0.9 for in-plate PPC-specific assay signal shows good correlation across all plates, indicating good control of run-to-run variation in signal intensity. The correlation of NC assay signal is less pronounced for nonspecific binding. Cells are shaded when correlation values are expressed elsewhere in the table or where the same antigen appears in the row and column.

### Cross-reactivity.

Estimation of cross-reactivity between assays within a well is a key element in multiplexed assay validation but was not addressed in our abbreviated protocol. Fortunately, the Democratic Republic of the Congo data set provides ample opportunity to assess this effect in the field and allows quantitation of nonspecific binding (NSB) for a given assay bead when all four of its partners are positive and for comparison with each assay bead when all 5 assays are negative (including tetanus [not discussed in this paper], *n* = 1,349). Measles presented a special condition with an *n* of 3, and so estimation includes all combinations where only three of its partners are positive, with *n* = 84 (8 mumps, 4 rubella, 45 VZV, and 27 tetanus). This was quantified by first taking the ratio of specific assay bead RLU to the average in-well negative bead RLU, which compensates for run-to-run variation in signal intensity, and then taking the average for each assay under each condition. As shown in Table 4, estimation of cross-reactivity, there is no discernible increase in NSB for any assay bead when all its partners within a well are positive.

**TABLE 4:**
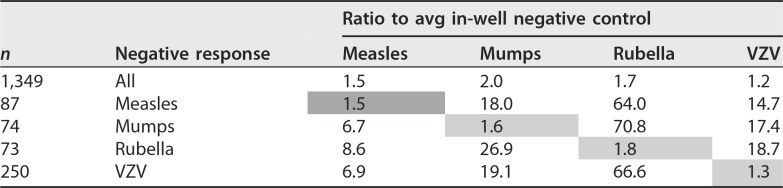
Estimation of cross-reactivity (ratio of average in-well negative control)^*a*^

a The data set was mined for samples positive for all assays but one, and signal intensity was compared to the average for the two in-well negative controls. Measles was a special case since only 3/10,000 samples satisfied this requirement; thus, all samples with 3/5 positive assays were used. When negative, the ratio for any given assay is not affected when all of its partners within a given well are positive. Shaded cells express cross-reactivity for the same antigen.

### Comparison of neat sera and DBS equivalent extract.

**FIG 5:**
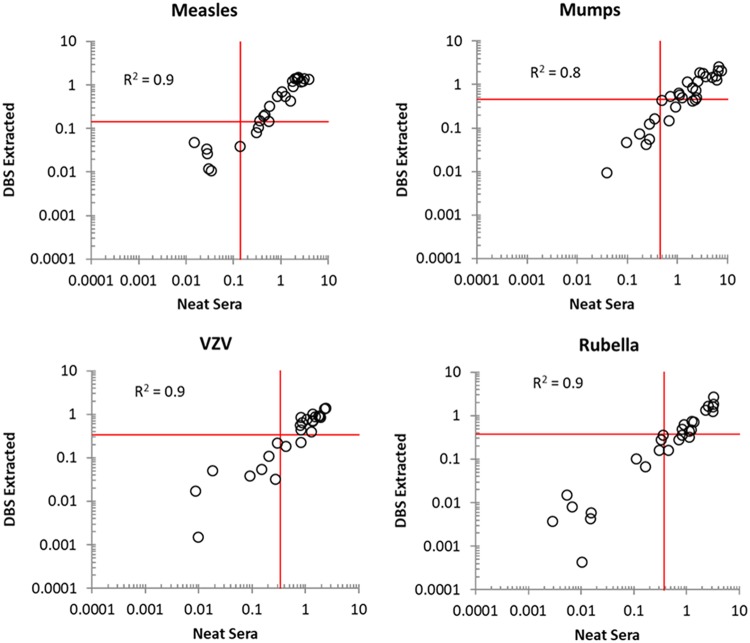
Regressions of neat sera versus dried blood spot (DBS) extract. In order to demonstrate equivalence between neat sera and extraction, DBS equivalents of the 32-member reference set were prepared, analyzed using the 54-well format, and compared to neat sera analyzed on the 96-well format. The red lines indicate seroprotecton cutoffs. All assays demonstrate 100% specificity as shown in Table 5, although there is a slight loss of sensitivity which may be the result of extraction inefficiencies.

DBS equivalents were prepared from our reference panel and independently extracted both in the Democratic Republic of the Congo and in the Dynex laboratory in Chantilly, VA. The specificity for all antigens tested both in the Democratic Republic of the Congo and at Dynex was 100%, while sensitivity ranged from 75.0% to 93.8%, with samples tested in the Democratic Republic of the Congo showing higher sensitivity overall, except for VZV, which demonstrated similar sensitivity (Table 5). Figure 5 shows regressions of neat sera versus extraction at both locations; for measles, rubella, and VZV, *R*^2^ was 0.90, while for mumps the value was a lower but adequate *R*^2^ = 0.80, further indicating high correlation between the neat sera and DBS equivalent samples.

**TABLE 5:** Sensitivity and specificity of neat sera versus DBS extracts^*a*^

Virus	Neat sera vs DBS by preparation site:			
	Dynex		DRC	
	% sensitivity	% specificity	% sensitivity	% specificity
Measles	90.9	100.0	91.7	100.0
Mumps	78.3	100.0	82.6	100.0
Rubella	75.0	100.0	93.8	100.0
VZV	88.2	100.0	88.2	100.0

a Sensitivity and specificity of neat sera were calculated using DBS extracts prepared independently at the Dynex laboratory and in the Democratic Republic of the Congo (DRC).

### Independent measles comparison with ELISA in Kinshasa, Democratic Republic of the Congo.

**FIG 6:**
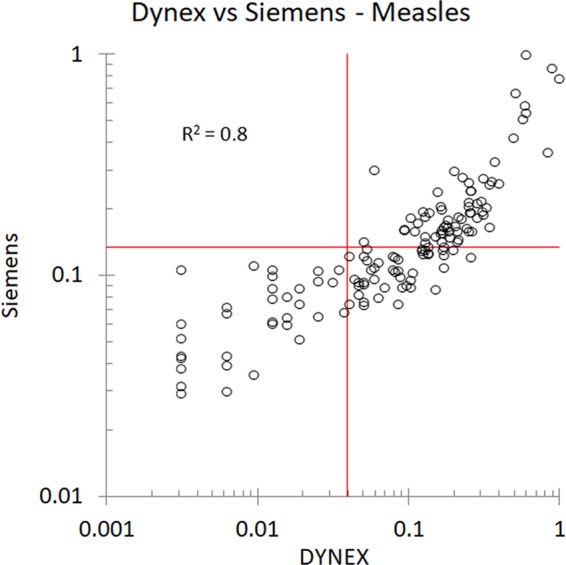
Comparison with Siemens ELISA anti-measles IgG and Dynex chemiluminescent multiplexed immunoassay. One hundred forty-four dried blood spots (DBS) were reextracted and tested independently using Siemens Enzygnost anti-measles virus IgG ELISA. There is good correlation between platforms as shown by an *R*^2^ of 0.8.

Dynex chemiluminescent multiplexed immunoassay measles results were compared with the Siemens ELISA kit. Figure 6 shows that the concordance between the two tests was quite strong, with *R*^2^ = 0.8.

## DISCUSSION

This report is novel in the use of DBSs and provides a practical and robust solution in resource-limited LMICs for the assessment of MMRV seroprevalence. The results of the Dynex chemiluminescent MMRV multiplexed immunoassay panel demonstrate functional equivalence to conventional ELISA with regard to sensitivity and specificity using DBSs and neat sera. These data indicate majority agreement between the Dynex MMRV chemiluminescent multiplexed immunoassay and conventional ELISAs for determination of antibody presence against four disease antigens in human blood samples. Sensitivity analysis further showed that, at most, differential treatment of these indeterminate results caused the Dynex immunoassay sensitivity calculation to fluctuate by 3.2% and the specificity calculation to fluctuate by 2.1%, indicating robustness of the assay’s binary call system.

Poor correspondence between these assay cutoffs and the international standard dilution series was found; thus, the only clinically relevant and defensible cutoff claims that we make are those generated by our correlations with the FDA-cleared predicates and reported as a qualitative assay response. However, these cutoffs exhibit good correlation with all four predicates and, when applied to the Democratic Republic of the Congo data set, performed as expected. Serial dilutions of international standards show excellent linearity and dynamic range, but the cutoff for measles as calculated by predicate correlations is 31.9 mIU, which is far below the accepted standard cutoff of 120 mIU. To definitively correlate international units (IU) with assay response as calculated in the DRC data set, the Dynex chemiluminescent multiplexed immunoassay should be correlated with the plaque reduction neutralization test (PRNT), which was outside the scope of this project. As described by Hatchette el al. (16), correlation between enzyme immunoassay and PRNT is typically very poor, with the best quantitation at >192 mIU, with an *R*^2^ of 0.62.

Compared to other measles platforms, the correlation with the Dynex chemiluminescent multiplexed immunoassay fell below an optimal *R*^2^ value of 0.9 with a range between 0.76 and 0.88, but the *R*^2^ observed between the FDA-cleared predicates themselves averages only 0.8 (data not shown). As the Siemens measles kit is commonly used in seroprevalence studies (25–27), additional testing compared the performance of the Dynex chemiluminescent multiplexed limit-of-detection immunoassay to this gold standard, resulting in high correlation of measles results across the two platforms. However, concerns were raised, as the limit of detection and the Siemens manufacturer-recommended cutoff for positivity are close. Therefore, many studies have included equivocal results as positives since the manufacturer recommendations for equivocal include some observations within the range of 120 mIU/ml, and up to 10% of samples may have equivocal results based on the manufacturer-recommended cutoff (between delta optical density [OD] of 0.1 and 0.2) (19, 27, 28).

While methodology developed by the CDC for DBS extraction was followed, the Siemens kit is calibrated for serum rather than DBSs. All Siemens ELISA adjusted results were higher than expected, indicating that over 97% of samples would be considered positive. This effect was especially pronounced in negative samples with low adjusted change in absorption optical density (Δ*A*), probably due to decreased signal-to-noise ratio. Therefore, we were not able to interpret the Siemens results compared directly to the Dynex results using the published conversion formula and cutoffs.

**FIG 7:**
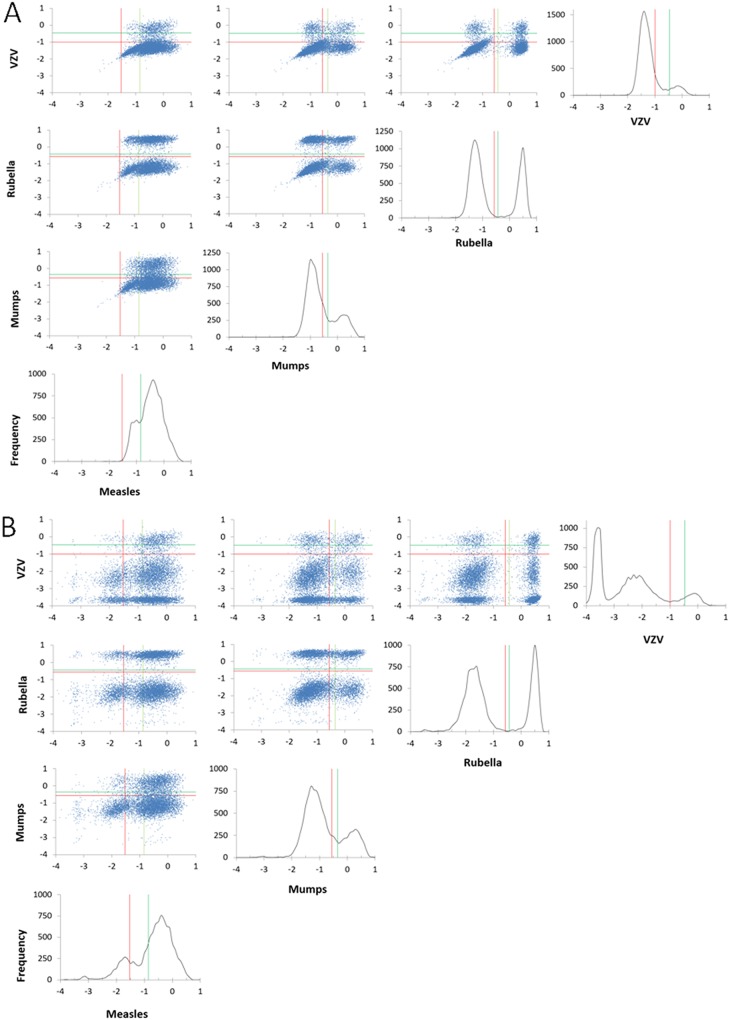
Full DRC data set (*n* = 9,778) showing assay score without subtracting nonspecific binding (A) versus score with subtraction (B) represented as histogram density plots and cross-correlations. These data clearly show improved discrimination of subpopulations via in-well negative bead subtraction and are a unique feature of the Dynex platform. The *y* axis of all density plots is frequency. All other axes are log(assay score). The green lines are upper seroprotection cutoffs, and the red lines are the lower cutoffs. Samples that fall between the two are considered indeterminate.

As shown through analysis of the PPC and NC extractions and through correlation of DBSs with neat sera, the system provides good accuracy and precision of the multiplex outcomes. A distinct advantage of this multiplexed system is the presence of negative-control beads within each well that allow for subtraction of NSB from each assay. As shown in Fig. 7, the effect is most pronounced for measles. The presence of negative-control assays within each well demonstrates that very little if any cross-reactivity can be observed, although NSB is evident. Further, intrawell negative controls allow for a higher level of discrimination between subpopulations via NSB subtraction. Variation was high and worthy of further examination but may reflect the resource-limited environment where the assays were performed. Uncontrolled variables include weekly conjugate preparations and water quality, which in turn affect extraction, sample incubation, and wash buffers.

While intrakit standardization and precision issues were observed, replication across multiple locations improves the confidence of these findings. However, in general, there remains a lack of standardization of multiplex assays across different kit types and manufacturers (29, 30). Variable protocols and operating procedures (especially with regard to dilution procedures, handling of DBSs versus sera, wash methods, and bead manufacture), as well as interlaboratory and interpersonal variability in execution of these protocols, all play a role in creating diverse multiplex assay results and need further evaluation. Finally, the determination of cutoff values for multiplex assays is somewhat controversial, and various methodologies and rationales exist, making it difficult to standardize the classification of antibody titer results as positive, negative, or indeterminate consistently across platforms. Optimization and normalization of results are key to increasing the clinical and public health utility of multiplex technologies and in lowering test result variance both across and within kits. There appears to be some loss in sensitivity when comparing neat sera and DBS extracts, possibly as a result of DBS extraction efficiency (Fig. 5).

Despite these limitations, multiplex assays present a promising platform for rapid analysis of high quantities of human blood samples, as may be collected in conjunction with national or regional serosurveys or during the investigation of population immunity during targeted epidemic events. Thanks to their utility in such large-scale projects, coupled with their low sample volume requirements, it is vital that the scientific community continue to undertake the validation of multiplex assays like the Dynex MMRV chemiluminescent assay, with special attention paid to their effectiveness in field settings using DBSs. Future studies should investigate the underlying causes of the disparities between the Dynex assays and traditional ELISA and work toward the creation of an international standard cutoff value for improved precision in serosurvey estimates. Ameliorating problematic cross-reactivity and other threats to multiplex validity and continuing to validate multiplex assay kits in a variety of field settings are essential to adoption and acceptance of these technologies for use in public health programming. Multiplex technology shows especially promising utility in the developing world, where the time, labor, and cost of running ELISAs can be prohibitive, and a dearth of trained laboratory personnel favors the adoption of automated testing procedures. The high throughput and short turnaround time of multianalyte testing on these devices also make them ideal for large-scale seroprevalence testing as part of surveillance activities and for evaluating vaccination programs by monitoring population immunity.

## MATERIALS AND METHODS

**FIG 8:**
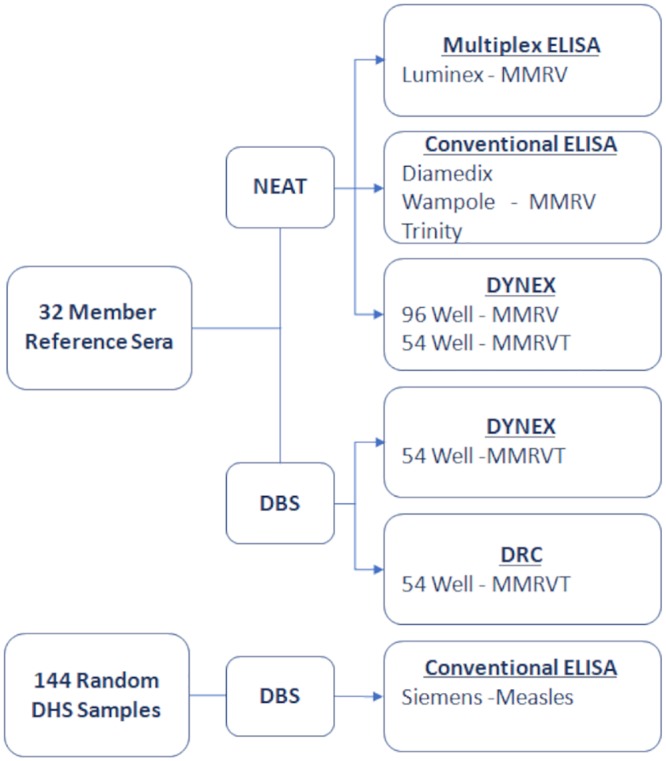
Abbreviated validation processing flow chart. A 32-member reference set of sera was selected to provide good coverage from high to low assay response across all assays. The reference set was processed as neat sera using one multiplex and three conventional ELISA kits and compared to the Dynex platform in both 96- and 54-well configurations. The reference set was prepared as dried blood spot (DBS) equivalents and processed independently in the Dynex laboratories and in the Democratic Republic of the Congo (DRC). One hundred forty-four DBSs were randomly selected from the DHS serosurvey and processed using a conventional Siemens ELISA kit.

To demonstrate the appropriateness and feasibility of this multiplex assay panel, we performed abbreviated validation studies as outlined in Fig. 8. Studies included comparative performance, including comparison to other FDA-cleared assays, of a 32-member reference panel both as neat serum and as DBS equivalents. Neat serum was tested on one commercial multiplex kit for MMRV and three different manufacturers’ kits for individual MMRV conventional ELISAs and compared to the Dynex chemiluminescent multiplex immunoassay. Reference panel DBS equivalents prepared using serum were further independently compared at the Dynex laboratory in Chantilly, VA, and at the INRB in Kinshasa, Democratic Republic of the Congo. A further comparison using 144 DBSs from the DHS was analyzed using the Centers for Disease Control and Prevention (CDC) gold standard widely accepted comparator ELISA for the detection of measles IgG antibodies (15).

### Assay formats.

**FIG 9:**
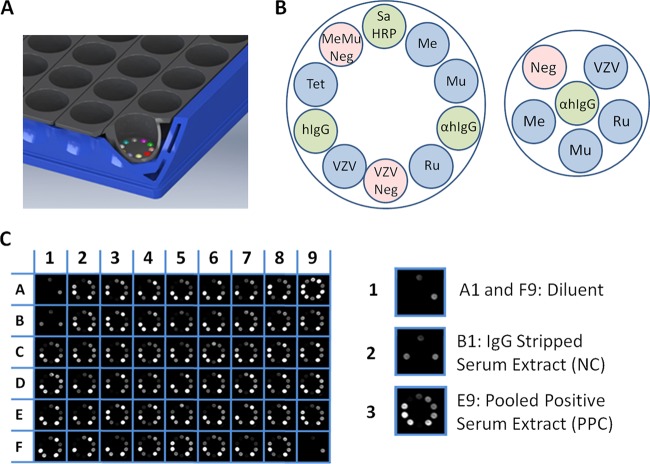
Dynex multiplexed assay plate detail and representative images. (A) Each assay well can contain up to 10 separate 2-mm polystyrene beads in the 54-well format, composed of 9 strips of 6 wells, or 6 beads in the 96-well format. (B) Fifty-four-well MMRVT and 96-well MMRV assay layouts. Blue circles represent specific assay beads (Me, measles; Mu, mumps; Ru, rubella; VZV, varicella-zoster; Tet, tetanus), green circles represent positive controls (Sa-HRP, streptavidin-horseradish peroxidase; αhIgG, goat anti-human IgG Fcγ; hIgG, total human IgG), and red circles represent the negative controls (VZV Neg, VZV negative; MeMu Neg, measles and mumps negative). The positive controls used in the 54-well format serve as processing controls, with all three being illuminated only when all assay steps have been properly carried out. (C) Representative image of a 54-well plate. Wells A1 and F9, diluent only; well B1, IgG-stripped serum extract (NC); well E9, pooled positive-control serum extract (PPC). All others have dried blood spot extracts. Inset 1 shows SA-HRP and hIgG positive-control beads being illuminated in response to diluent-only sample; inset 2 shows NC illuminating SA-HRP, hIgG, and anti-hIgG; inset 3 shows PPC illuminating all assays except negative-control beads.

As shown in Fig. 9, multiplex assay plates consist of a 96-well format using 12 separate 8-well 6-bead strips and a 54-well format using 9 separate 6-well 10-bead strips. Both plate layouts were used in these studies, a 54-well MMRVT panel including three positive and two negative controls and a 96-well MMRV panel including single positive and negative controls. The assembled plates were processed using a prototype Dynex Multiplier chemiluminescent automated immunoassay instrument, and images were captured and processed via a standalone, laptop-controlled charge-coupled device (CCD) camera (STF-8300; Diffraction Limited/SBIG, Ottawa, ON, Canada). All data were exported to and tabulated in Microsoft Excel.

### Processing reagents.

DBS extraction buffer was wash buffer consisting of phosphate-buffered saline (PBS) (Fisher BP661-10) and 0.05% Tween 20 (Fisher BP337-100) brought to 5% biotin-free dried milk (Lab Scientific M0841). The detection antibody was goat anti-human IgG Fcγ-specific horseradish peroxidase (HRP) conjugate (Jackson Immunoresearch 109-035-098) reconstituted to 1 mg/ml in Stabilzyme HRP conjugate stabilizer (SurModics SZ02) and diluted to either a 1:200,000 (Dynex) or a 1:400,000 (Democratic Republic of the Congo) working stock in synthetic blocking buffer (SurModics STSB). Chemiluminescent luminol HRP substrate was Michigan Diagnostics SHRPE21008DT. The detection reagent used in the Democratic Republic of the Congo was prediluted to 1:100 in Pierce peroxidase conjugate stabilizer (Pierce 31503) and stored at −20°C until use. A blend of 5 healthy North American donors providing a robust signal for each assay served as pooled positive control (PPC) and were prepared as DBS equivalents. These were extracted and run on every plate and assay response for each sample calculated as a ratio to this control. The negative control (NC) came from pooled IgG-stripped normal serum (BioIVT, Westbury, NY), likewise prepared as DBS equivalents, which were extracted and run on each plate. Each lot of beads was assessed for activity via (i) a dilution series of PPC to generate assay response curves and (ii) a checkerboard pattern of IgG-stripped negative-control sera and a middle of the dynamic range (mid-cal) dilution of PPC (data not shown).

### MMRV assay and control beads.

Assay beads were 2-mm 220 grit polystyrene beads (Tsubaki Nakashima, Sault St. Marie, MI) irradiated at 65 to 85 kGy. Coating was carried out in 10 mM NaCO_3_ (pH 9.0) at 50 μl/bead, rolling overnight at room temperature. Antigen concentrations were as follows: for measles, 10 μg/ml (NF0608; Ross Southern Labs, Spanish Fork, UT); mumps, a blend of Jeryl Lyn (Ross NF0708) and Enders (Ross NF070804), each at 5 μg/ml; rubella, 5 μg/ml (6076; Meridian, Memphis, TN); and VZV, 5 μg/ml (Ross NF0508). Positive-control bead 1 (SA-HRP bead), which is illuminated in the presence of luminol-HRP substrate alone, was coated sequentially with 5 μg/ml streptavidin (016-000-114; Jackson Immunoresearch) followed by 5 μg/ml biotinylated HRP (29139; Pierce). Positive-control bead 2 (hIgG bead), which is illuminated with anti-human HRP conjugate followed by luminol, was coated with 200 ng/ml total human IgG (U.S. Biologicals I1903-90S). Positive-control bead 3 (anti-hIgG bead), which is illuminated only when all assay steps using human sera have been carried out, was goat anti-human IgG Fcγ specifically coated at 0.4 μg/ml (Jackson Immunoresearch 109-005-098). Negative-control bead 1 (MeMu Neg) for measles and mumps was MRC-5 cell lysate at 5 μg/ml (Ross NF0601), and negative-control bead 2 (VZV Neg) for VZV was E6 cell lysate at 5 μg/ml (Ross NF0501). All beads were blocked for 30 min with Stabilguard Choice (SurModics SG02) and dried desiccated overnight before being assembled into plates (Fig. 9). Once assembled, all plates were stored desiccated and heat sealed in Mylar pouches at 4°C until use.

### Plate processing.

All steps using the 10-bead 54-well format used 250 μl/well, while the 6-bead 96-well format used 100 μl/well. Once applied to the plate, samples were incubated at room temperature, agitated for 1 h, and then washed. Detection antibody was applied and was incubated and agitated for 30 min. Equal volumes of luminol parts A and B were applied, incubated, and agitated for 2 min. The plate was visualized by the CCD camera using a 60-s exposure. Relative light units (RLU) were calculated via ArrayPro image processing software (Media Cybernetics, Rockville, MD), and data were analyzed using Microsoft Excel.

### International standards and linearity.

**FIG 10:**
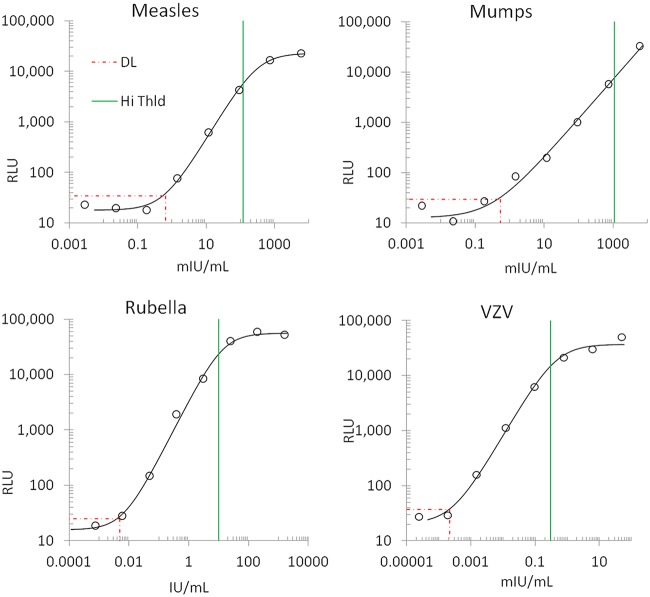
4PL fit of international dilution standards. NIBSC international standards for measles, rubella, and VZV were reconstituted according to the manufacturer’s instructions, gravimetric serial dilutions were measured as relative light units (RLU; open circles), and a 4PL fit was calculated (solid line) using the equation *y* = (*d* − *a*)/[1 + (*x*/*c*)*^b^*], where *a* = bottom, *b* = Hill’s slope, *c* = midpoint, and *d* = top of the curve. Average linearity (Hill’s slope) is 1.02 ± 0.06. Detection limits (DL; red dotted lines) represent 2.5 SDs over system noise. High threshold (green line) represents the relative seroprotection cutoff (Table 1).

International standards used were measles (National Institute for Biological Standards and Control [NIBSC] 97/648), rubella (NIBSC RUBI-1-94), and VZV (NIBSC W1044) (Table 1). An international standard for mumps was unavailable, and so, assay response to the measles standard was used as a proxy. Each standard was gravimetrically reconstituted according to the manufacturer’s instructions, serial gravimetric dilutions were made into SurModics BioFX sample diluents, and all assays demonstrate ∼4 logs of dynamic range. Linearity was calculated via 4-parameter logistic (4PL) fit. Limits of detection represent signal 2.5 standard deviations above assay noise (Fig. 10).

### Comparison to conventional ELISA.

To estimate assay performance between platforms and to establish cutoffs, comparisons were carried out analyzing the 32-member reference set using predicate commercial assay kits shown in Table 6. All conventional ELISA kits were processed on a Dynex DS2 automated processing instrument according to the manufacturer’s instructions using vendor-validated protocols. The Luminex multiplexed kit was processed on a Luminex D200. A 32-member reference panel was selected from >100 normal human sera from commercial sources that span low to high reactivity for MMRV. Cutoffs were calculated as the assay response corresponding to a best fit versus all predicates. Seroprotection levels are expressed as ratio of signal to PPC and summarized along with the limit of quantitation (LOQ) in Table 1.

**TABLE 6:** Commercially available kits used for comparative study

ELISA vendor	Catalog no. or name			
	Measles	Mumps	Rubella	VZV
Zeus/Luminex	A93101G/A93111G	A93101G/A93111G	A93101G/A93111G	A93101G/A93111G
Wampole	X426000CE	X425900CE	X425300CE	X425600CE
Diamedix	720-520	720-540	720-360	720-380
Trinity	2326000	2325900	2325300	2325600
Siemens	Enzygnost anti-measles virus IgG			

### Dried blood spot equivalents.

DBS equivalents were prepared from the 32-member reference set via application of 30 μl serum to Whatman 903 Protein Saver cards (Whatman Z761575) and handled according to the manufacturer’s instructions. Extractions were carried out according to the work of Mercader et al. by placing a single 0.25-in. punch in 1 ml PBS (Fisher BP661-10)–0.01% Tween 20 (Fisher BP337-100)–5% biotin-free dried milk (Lab Scientific M0841) in a Parafilm-covered round-bottomed 12- by 75-mm tube and shaking the tube at room temperature for 1 h (19). This represents a 1:143× dilution, assuming 7 μl of whole blood per 0.25-in. punch. Neat sera were processed at a 1:20 dilution into SurModics BioFX.

### Neat sera versus DBS equivalent extracts.

The 32-member reference set was processed on 54-well plates as neat sera at the Dynex laboratory, and DBS equivalent extracts were independently prepared and processed in both the Dynex and the Democratic Republic of the Congo laboratories. DBSs were extracted independently in the Dynex laboratory in Chantilly, VA, and in the INRB laboratory in Kinshasa, Democratic Republic of the Congo, and comparisons were made using assay response. Sensitivity and specificity were calculated assuming that all positive and intermediate Dynex multiplexed assay results from neat sera were true positives and all negative calls were true negatives.

### Independent measles comparison with ELISA in Kinshasa, Democratic Republic of the Congo.

One hundred forty-four DBS specimens were randomly selected and tested from the DRC-DHS in the Democratic Republic of the Congo. Comparison was completed using the Siemens ELISA measles kit. Sample reconstitution followed the CDC protocol for DBS reconstitution for the ELISA kit (19). Briefly, samples were reconstituted from each DBS with PBS, according to Dynex protocol. Two 0.25-in. punches were taken from each DBS collected on Whatman 903 filter paper, placed in a tube with 1 ml of PBS, and shaken for 1 h at 0.75× maximum speed. These aliquots were then further diluted in the ELISA and multiplex sample preparations, according to their respective procedures described below. Each DBS punch was assumed to contain the equivalent of 6 μl of serum (31).

### Data analysis.

All data analyses were performed using Microsoft Excel software. Binary positive/negative calls were made for each sample’s test result based on the specific cutoffs described above for each individual assay. In order to complete a full comparison of the Dynex MMRV IgG assay with ELISA, both percent agreement and Cohen’s kappa coefficients were calculated. Where not otherwise noted, indeterminate results have been assigned a positive call.

### Ethics.

The parent or guardian of each child enrolled in the serosurvey provided consent on their behalf, since children fell below the standard age for consent. Institutional review board approval was obtained at UCLA Fielding School of Public Health, Los Angeles, CA; the Kinshasa School of Public Health, Kinshasa, Democratic Republic of the Congo; and the CDC, Atlanta, GA.

### Data availability.

Data files associated with this report can be accessed via the Dryad Digital Repository (datadryad.org, https://doi.org/10.5061/dryad.8br4n80) and include (i) MMRV IU calibration curves, a dilution series of international standards run on the Dynex platform; (ii) predicate comparisons, the 32-member reference set run in duplicate on the Dynex platform versus Wampole, Diamedix, Trinity, and Luminex immunoassays; (iii) DBS versus neat sera, comparing the 32-member reference set run on the Dynex platform as neat sera and DBS equivalents; and (iv) 2013 DRC-DHS Survey Data.csv, the full DRC-DHS set listed by plate run.
